# Modelling Compression Strength of Waste PET and SCM Blended Cementitious Grout Using Hybrid of LSSVM Models

**DOI:** 10.3390/ma15155242

**Published:** 2022-07-29

**Authors:** Kaffayatullah Khan, Jitendra Gudainiyan, Mudassir Iqbal, Arshad Jamal, Muhammad Nasir Amin, Ibrahim Mohammed, Majdi Adel Al-Faiad, Abdullah M. Abu-Arab

**Affiliations:** 1 Department of Civil and Environmental Engineering, College of Engineering, King Faisal University, P.O. Box 380, Al-Hofuf 31982, Saudi Arabia; mgadir@kfu.edu.sa (M.N.A.); malfaiad@kfu.edu.sa (M.A.A.-F.); 219041496@student.kfu.edu.sa (A.M.A.-A.); 2 Department of Civil Engineering, GLA University, Mathura 281 406, India; jitendra.gudainiyan@gla.ac.in; 3 Department of Civil Engineering, University of Engineering and Technology, Peshawar 25120, Pakistan; mudassiriqbal@uetpeshawar.edu.pk; 4 Transportation and Traffic Engineering Department, College of Engineering, Imam Abdulrahman BinFaisal University, P.O. Box 1982, Dammam 31451, Saudi Arabia; ajjamal@iau.edu.sa; 5 Applied Research Center for Metrology Standards and Testing, Research and Innovation, King Fahd University of Petroleum and Minerals, Dhahran 31261, Saudi Arabia; ibrahim@kfupm.edu.sa

**Keywords:** cementitious grouts, polyethylene terephthalate waste, supplementary cementitious materials, swarm intelligence, particle swarm optimization

## Abstract

Nowadays, concretes blended with pozzolanic additives such as fly ash (FA), silica fume (SF), slag, etc., are often used in construction practices. The utilization of pozzolanic additives and industrial by-products in concrete and grouting materials has an important role in reducing the Portland cement usage, the CO_2_ emissions, and disposal issues. Thus, the goal of the present work is to estimate the compressive strength (CS) of polyethylene terephthalate (PET) and two supplementary cementitious materials (SCMs), namely FA and SF, blended cementitious grouts to produce green mix. For this purpose, five hybrid least-square support vector machine (LSSVM) models were constructed using swarm intelligence algorithms, including particle swarm optimization, grey wolf optimizer, salp swarm algorithm, Harris hawks optimization, and slime mold algorithm. To construct and validate the developed hybrid models, a sum of 156 samples were generated in the lab with varying percentages of PET and SCM. To estimate the CS, five influencing parameters, namely PET, SCM, FLOW, 1-day CS (CS_1D_), and 7-day CS (CS_7D_), were considered. The performance of the developed models was assessed in terms of multiple performance indices. Based on the results, the proposed LSSVM-PSO (a hybrid model of LSSVM and particle swarm optimization) was determined to be the best performing model with R^2^ = 0.9708, RMSE = 0.0424, and total score = 40 in the validation phase. The results of sensitivity analysis demonstrate that all the input parameters substantially impact the 28-day CS (CS_28D_) of cementitious grouts. Among them, the CS_7D_ has the most significant effect. From the experimental results, it can be deduced that PET/SCM has no detrimental impact on CS_28D_ of cementitious grouts, making PET a viable alternative for generating sustainable and green concrete. In addition, the proposed LSSVM-PSO model can be utilized as a novel alternative for estimating the CS of cementitious grouts, which will aid engineers during the design phase of civil engineering projects.

## 1. Introduction

In recent times, plastic manufacture and increased use have placed a tremendous environmental strain on society [1]. The production of plastic waste is fast expanding in tandem with population growth, and it is estimated that by 2050, global plastic waste production will have doubled [2]. Plastic production expanded exponentially between 1950 and 2015, from 2.3 million tons to 448 million tons [3]. According to the literature, over half of all plastic has been manufactured in the last 15 years [4]. Approximately 8 million tons of plastic garbage are washed into the oceans yearly [5]. Since the 1950s, more than 8.3 billion tons of plastic has been created, with around 60% of that plastic ending up in landfills and the natural environment [6]. Thus, it is essential to investigate the recycling of plastic wastes to address this global environmental challenge. It is also desirable to substitute virgin materials used in construction with waste resources. Therefore, recycling plastic waste in construction projects can help achieve this goal to a large extent. Moreover, recycling waste plastic on a large scale can substantially contribute to the economy and environment. In contrast, the consumption of natural resources can be decreased by substituting plastic waste for aggregates or sand, thereby reducing environmental pollution and construction costs, and enhancing the engineering qualities of asphalt concrete. Furthermore, recycling plastic waste can lessen the burden of landfills and conserve marine life. Due to population expansion and industrialization, industrial wastes and/or by-products (such as FA and SF) which pose a grave threat to the ecosystem are increasing day by day. Therefore, the reuse and recycling of these wastes and/or by-products could be a feasible alternative in the construction industry.

In construction work, waste plastic has frequently been used as a bitumen or aggregate replacement [7]. Significant improvements in the performance attributes of binder and asphalt mixtures has been shown in the literature [8]. To date, very little research has been undertaken to investigate the use of polyethylene terephthalate (PET) in cementitious materials [9,10]. Furthermore, the impact of PET on the mechanical properties of cementitious materials has been explored [11]. Note that waste plastics are utilized as a fiber or a replacement for fine and coarse aggregates in the concrete industry [12]. Generally, waste plastics are used to make lightweight concrete elements by replacing aggregates [13]. According to a recent study, incorporating recycled PET as a fiber in the concrete mixture increased the tensile strength and crack resistance [14]. It is pertinent to mention here that with the incorporation of PET as an aggregate or sand replacement with a volume greater than 10%, the compressive strength (CS) of concretes is significantly reduced compared to standard strength of concrete. Partial replacement of cement with plastic waste was also investigated in the literature, and a considerable drop in CS was found [15]. Specifically, a 5% to 20% substitution of cement can reduce the concrete CS by between 23% and 72% [16]. Schaefer et al. [17] noticed a reduction in CS when waste PET was utilized as a partial replacement for cement in mortars. It is interesting to note that, according to Schaefer et al. [17], using irradiated plastic (by subjecting it to gamma radiations) in place of conventional plastic can partially restore the strength loss of concrete caused by the addition of plastic. PET is a semi-crystalline polyester that has an isotropic microstructure because to its glassy amorphous nature [18]. Irradiation has an impact on the two key properties of PET, namely chain-scission and crosslinking. Due to the chain-scission action, PET crystallinity rises [19]. Irradiation-induced crosslinking of the polymers results in improved adhesion, toughness, material stability, and high impact resistance [20]. Gamma radiation aids in enhancing the concrete’s performance characteristics. Fiber-reinforced concrete (FRC) has considerably enhanced the physical characteristics of cementitious composites. Exposure to gamma radiation can further enhance the mechanical characteristics of FRC [21]. This results from the polymer’s structure being altered by irradiation through the processes of crosslinking, scission, and chain grafting, which are all dependent on the gamma dosage that was administered [22]. Schaefer et al. [17] recently investigated the possibility of using irradiated plastic in place of cement to create a cementitious composite. In contrast to non-irradiated plastic, they discovered that plastic exposed to radiation had improved mechanical characteristics and a compact microstructure. Further investigation is necessary to determine the impact of irradiation plastic alone or in conjunction with commercially available silica fume on the initial and hardened characteristics of a cementitious composite. This will improve the researchers’ comprehension of the possible application of radioactive plastic as a cement replacement in the building sector. The strength of cement mortar can be increased by substituting gamma-irradiated PET with additional replacement of fly ash (FA) or silica fume (SF) [9]. A detailed review of the literature shows that the addition of gamma-irradiated waste plastic to cementitious grouts can improve the strength of cementitious grouts [23].

FA and SF are classified as supplemental cementing materials (SCM). Numerous studies have been conducted on the effects of mineral admixtures, such as FA and SF, on the mechanical characteristics of geopolymer and Portland cement concrete [24,25]. Golewski [26] discovered that FA might lengthen the concrete’s real fracture path, increasing the concrete’s durability. According to Gil et al. [27], the simultaneous application of FA and SF modifies the concrete’s fracture toughness based on the Mode I fracture. It could be possible to make concrete using siliceous FA and SF instead of cement. Cementitious composites with higher alumina content mineral admixtures, such as metakaolin, ceramic waste powder, and clay brick waste powder, showed greater compressive strength at high temperatures [28]. According to Khan et al. [29,30], FA, SF, and calcium carbonate whisker may significantly enhance the mechanical characteristics of fiber-reinforced concrete. FA and SF can improve the performance of cement concrete due to their pozzolanic properties. In order to achieve sustainability, greater performance, and economical benefits, the use of materials or mineral admixtures to substitute cement in mortar and concrete is almost unavoidable. Therefore, recycling municipal and industrial wastes or waste by-products as a replacement for cement, sand, or aggregates could be beneficial to the environment in terms of reducing the use of non-renewable natural resources. This will also be advantageous to the construction industry in terms of cost savings and enhanced concrete qualities. On the contrary, the CS of cementitious grout is typically determined in the laboratory [31]. The determination of the CS of cementitious grout in the laboratory is time-consuming and costly. One of the causes is the need for specialized laboratory equipment. In addition, professional engineers and highly skilled technicians are required for manufacturing and testing. Hence, it is of practical need to come up with intelligent data-driven methods to determine the CS of cementitious grout based on existing test results.

Currently, soft computing techniques are gaining popularity due to their superior predictive abilities compared to regression-based techniques, and they are used to mimic the complex behavior of a variety of structural engineering problems [32,33,34,35]. In civil engineering, the application of machine learning (ML) algorithms has been extensively documented since 2000 [36]. In recent decades, numerous ML algorithms, such as artificial neural networks (ANNs) [37,38], support vector machine (SVM) [39], least-square support vector machine (LSSVM) [40], genetic programming (GP) [41], gene expression programming (GEP) [42], extreme learning machine (ELM) [43], multivariate adaptive regression spline (MARS) [44], adaptive neuro-fuzzy inference system (ANFIS) [45], and more [46,47], have been utilized to solve a variety of engineering problems.

Ferreira and Jalali [48] estimated the early age of CS using a strategy based on non-destructive testing results. Ni and Wang [49] used multi-layer feed-forward neural networks to predict the 28-day CS (CS_28D_) of concrete. Rafi and Nasir [50] proposed an analytical method for forecasting CS_28D_ based on the 7-day CS (CS_7D_) of concrete. Despite their practical significance, prior researchers have made no attempts to build regression-based ML models for estimating the CS_28D_ of waste PET/SCM mixed cementitious grout. Thus, this study was motivated to fill the gap in the literature. Specifically, LSSVM was used to compute the CS_28D_ of waste PET/SCM blended cementitious grout.

Based on the most recent research, it has been determined that ML approaches are ideally suited for predicting the CS of concrete. In addition, as the topic of interest is complex, it is necessary to examine various sophisticated ML models in order to identify more accurate estimating models. The LSSVM is an effective instrument for nonlinear and multivariable modelling [51]. This regression-based ML model has been effectively implemented in different engineering domains. However, none of the prior research has used hybrid LSSVM models to estimate the CS_28D_ of waste PET/SCM blended cementitious grout. Therefore, the purpose of the present study is to fill this gap in the literature.

It is important to note that the LSSVM model requires proper configuration of its hyperparameters, including the regularization (γ) and kernel function (σ) parameters. These two hyper-parameters have a substantial impact on the outcome of the learning phase and, consequently, influence the predictive ability of the LSSVM-based model. Note that it is not so easy to specify these parameters since they must be sought in continuous domains and there is an infinite number of parameter sets. Therefore, numerous researchers have combined metaheuristic (MH) algorithms and ML models because parameter tuning problems can be phrased as optimization problems.

Previous studies have demonstrated the efficacy of MH algorithms in modelling complex phenomena in different engineering problems. Combining symbiotic organisms search (SOS) and LSSVM, Prayogo and Susanto [52] improved the predictive accuracy of the employed models to calculate the friction capacity of driven piles in cohesive soil. Yuan et al. [53] used LSSVM–GSA (a hybrid model of LSSVM and gravitational search algorithm) to estimate the short-term wind power. Xue [54] presented a hybrid LSSVM model of particle swarm optimization to predict slope stability. Nevertheless, the application of swarm intelligence (SI) algorithms in CS estimation of PET/SCM blended cementitious grout has not yet been investigated. Therefore, this study presents a comparative assessment of five hybrid LSSVM models constructed with five distinct SI algorithms, namely particle swarm optimization (PSO), grey wolf optimizer (GWO), salp swarm algorithm (SSA), Harris hawks optimization (HHO), and slime mold algorithm (SMA), to optimize the hyper-parameters of LSSVM [55].

## 2. Materials and Methods

For developing hybrid models, the data was obtained from the previous experimental results of Khan et al. [9], previously employed by Khan et al. [56] for developing genetic programming models. Ordinary Portland cement (OPC), PET waste, SF, FA, and superplasticizer were obtained for the experimental work. The PET waste with a particle size of less than 150 µm was used as a substitute for cement.

The cementitious grouts were mixed in the laboratory as per the ASTM provision [57]. The required quantity of cement, PET waste, FA, and SF were initially dry mixed followed by adding two-thirds of water and further mixing. Note that, to ensure the homogeneity of cement grouts, the remaining water and superplasticizer were added and blended thoroughly. After mixing, flow cone apparatus was used to the flow the freshly prepared cement grout [9]. According to ASTM specifications, 1 L of fresh grout must flow out of a cone in 11 to 16 s [58]. All combinations of grouts and their CS was evaluated using a compression testing machine. In each case, 50 mm × 50 mm × 50 mm mould was used. The CS of cement grouts was tested after 1-day, 7-day, and 28-day curing. For this purpose, a universal testing machine of 3000 kN capacity was used. Subsequently, the 1-day, 7-day, and 28-day CS (i.e., CS_1D_, CS_7D_, and CS_28D_, respectively) of hardened cement grouts were recorded [59].

### 2.1. Computational Approaches

The working principle of LSSVM as well as a brief overview of MH algorithms are presented and discussed in this sub-section.

#### 2.1.1. Least-Square Support Vector Machine (LSSVM)

LSSVM, a regression-based ML approach based on the structural risk reduction principle, was introduced by Suykens et al. [60]. The learning phase of LSSVM is rapid because it only entails solving a set of linear equations., The dataset can be prepared in the following format to build a prediction model in LSSVM: $D=\left\{x_{k},y_{k}\right\}$, k=1, 2,…, N; where k is the kth sample and N is the total number of samples. LSSVM tries to develop a mapping function y(x) that estimates the response variable against a set of input parameters x. In LSSVM, the following formulation is used for function approximation.

(1) $$y\left(x\right)=\overset{N}{\underset{k=1}{\sum }}\alpha_{k}K\left(x_{k},x_{l}\right)+b$$

where k is the index number; b is the bias; and $K\left(x_{k},x_{l}\right)$. is the kernel function. Generally, the radial basis function is used kernel function, which can be given by:

(2) $$k\left(x_{k},x_{l}\right)=\exp\left(-\frac{{\left|\left|x_{k}-x_{l}\right|\right|}^{2}}{2\sigma^{2}}\right)$$

where σ. is the kernel parameter. The following optimization task is necessary to construct a LSSVM model:

(3) $$J_{P}\left(w,e\right)=\left\{\frac{1}{2}w^{T}w+\frac{\gamma }{2}\overset{N}{\underset{k=1}{\sum }}e_{k}^{2}\right\},$$

(4) $$y_{k}=w^{T}\emptyset \left(x_{k}\right)+b+e_{k}$$

where $e_{k}\in R$ represents the kth error variable; w and b are the two parameters that are used function approximation; γ and $\emptyset \left(x_{k}\right)$ are the regularization constant and mapping function, respectively.

#### 2.1.2. Overview of MH Algorithms

**Figure 1:**
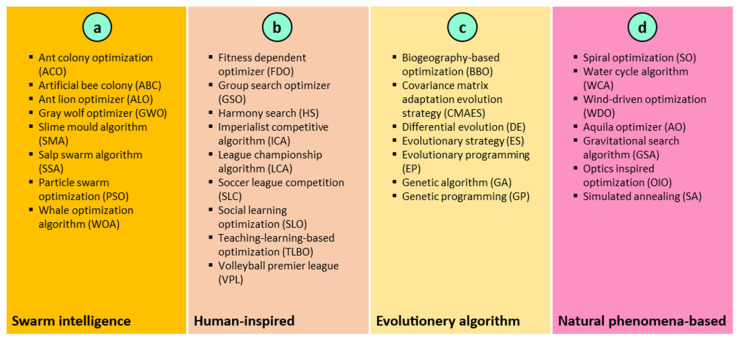
(**a**–**d**) Classification of MH algorithms.

This sub-section provides a brief description of MH algorithms. In general, the adoption of MH approaches to solve various problems has increased tremendously [61]. They are gradient methods that are free and can tackle highly complicated optimization problems more effectively than conventional approaches [62]. Additionally, they are easier to execute and more efficient than conventional optimization techniques. There are various inspirations for MH approaches, which can be categorized into four groups. These categories include (a) swarm intelligence (SI) techniques, (b) human-inspired algorithms, (c) evolutionary algorithms (EAs), and (d) natural phenomenon approaches. Figure 1 illustrates these categories.

The first group, referred to as SI techniques, mimics the behavior of swarms in nature during food-seeking. ACO [63], ABC [64], ALO [65], GWO [66], SMA [67], SSA [68], PSO [69], and WOA [70] are the most common algorithms in this group. The second group is reliant on human behavior. The human-inspired algorithms are FDO [71], GSO [72], HS [73], ICA [74], LCA [75], SLC [76], SLO [77], TLBO [78], and VPL [79]. The motivation for the algorithms in the third group, known as EAs, comes from mimicking natural genetic concepts such as crossover, mutation, and selection. This category includes a number of MH techniques such as BBO [80], CMAES [81], DE [82], ES [83], EP [84], GA [41], and GP [85]. The fourth group is attempting to imitate natural phenomena such as rain, spirals, wind, and light. This group includes SO [86], WCA [87], WDO [88], AO [89], GSA [90], and SA [91] are some of the other MH approaches that are based on physical laws.

In this work, five SI algorithms including PSO, GWO, SSA, HHO, and SMA were used to optimize the hyper-parameters of LSSVM and five hybrid LSSVM models, namely LSSVM-PSO, LSSVM-GWO, LSSVM-SSA, LSSVM-HHO, and LSSVM-SMA, were constructed. Note that detailed working principles of the employed MH are not presented in this study because they have already been established in the literature. The original studies of Kennedy and Eberhart [69] for PSO, Mirjalili et al. [66] for GWO, Mirjalili et al. [68] for SSA, Heidari et al. [92] for Harris hawks optimization (HHO), and Li et al. [67] for SMA, can be referred to for this purpose. However, a brief overview of these MH is presented in the following sub-section.

#### 2.1.3. A Brief Overview of Employed MH Algorithms

In this sub-section a brief overview of the employed algorithms, namely PSO, GWO, SSA, HHO, and SMA, is presented.

PSO [69,93] is a swarm-based MH inspired by the motion of bird flocks and schooling fish. The fundamental goal of this MH is to find the globally-optimal solution using a multidimensional search space. PSO begins its search operation by initializing the random velocities and locations of the particles. In PSO, each particle moves based on its “best” position and the “best” position of the group as a whole, but they tend to move randomly. The particle’s position is updated according to its own best position and the direction of the global best position. Iteratively, the particle velocities are updated based on the difference between their personal best position and the global best location. Utilizing exploitation and exploration processes, the particles eventually converge on the ideal solution.

Mirjalili et al. [66] introduced GWO based on the hunting behavior of grey wolves. In the wild, grey wolves live in groups of 5 to 12 individuals. Based on their responsibilities and decision-making roles during prey hunting, they are categorized into four types: (a) the group leader is the alpha (α) wolf, (b) the second-in-command is the beta (β) wolf, (c) the subordinate is the delta (δ) wolf, and (d) the lowest ranking is the omega (ω) wolf. In GWO, individuals (referred to as solutions) are ranked from best to worst after being evaluated using an objective function. The hunting process can be divided into three stages: (a) tracking, chasing, and approaching the prey, (b) pursuing, encircling, and attacking the prey, and (c) attacking the prey. Exploration takes place in the first two stages, with exploitation taking place in the final stage.

In SSA [68], salps are members of the Salpidae family and have a cylinder-shaped, translucent body. Their tissues also resemble jellyfish closely. Moreover, salps and jellyfish have a locomotory system in which water pushed into the body propels them forward. In SSA, the population of salps is initially divided into two categories, namely leaders and followers. The position of the leader is at the beginning of the chain, while the remainder of the chain consists of followers. As with other SI approaches, the location of the salps is specified in an n-dimensional search space. Consequently, the positions of all salps are entered into a two-dimensional matrix. The SSA algorithm is good at exploitation and exploration processes, which are both essential for avoiding local optima and discovering improved solutions. During exploration, the entire search space is examined to determine the potential locations of a solution. In order to find the optimal solution, these areas are exhaustively examined during the exploitation process.

HHO, as proposed by Heidari et al. [92], is based on Harris hawks’ cooperative treatment and pursuit behavior. In HHO, hawks descend from several directions in an attempt to startle their prey. In addition, Harris hawks are able to select a certain method of pursuit based on the battle patterns of their target. HHO consists of three fundamental stages: (a) amaze pounce, (b) tracking the target, and (c) many striking approaches. The first phase is called exploration and is designed to wait, search, and mathematically discover the desired hunt. The second phase transitions from exploration to exploitation and is completed based on a rabbit’s external energy. In the final phase (known as exploitation), hawks frequently assume a soft and sometimes hard environment to chase rabbits from multiple directions. As a global optimizer, HHO has the benefit of being able to handle issues with constraints.

In 2020, Li et al. [67] introduced SMA as one of the nature-inspired MH algorithms. It is a mathematical model for simulating the propagation of slime mold waves. Due to their unique characteristics and pattern, slime molds are able to utilize multiple food sources simultaneously, allowing them to develop a venous network for their connections. In light of these positive and negative responses, slime may be the most effective strategy for food connection. SMA adaptively replicates the process of providing negative and positive feedback during the propagation wave. Potentially, slime molds could adjust their search patterns dynamically based on the quality of the food origin. SMA consists of two major levels: (a) obtaining food in the manner in which slime collects food based on its scent in the air, and (b) warping food in which slime conducts venous configuration contraction. Due to higher exploitation and exploration abilities, SMA has been used in solving numerous optimization tasks,

## 3. Dataset and Modelling

This section presents and discusses the descriptive details of the experimental results. In addition, the computational approach for building hybrid LSSVM models is presented.

### 3.1. Descriptive Details

The descriptive details of the input (PET, SCM, FLOW, CS_1D_, and CS_7D_) and output (CS_28D_) parameters are tabulated in Table 1. Herein, the minimum, mean, median, mode, range, and the maximum values, standard error, standard deviation, sample variance, Kurtosis, and skewness for all the parameters are presented. A smaller standard error (in the range of 0.29 to 0.90) implies that the experimental database is quite trustworthy. Moreover, the sample variance, which is between 13.55 and 126.89, demonstrates the presence of a diverse set of test results. The parameters PET, SF, CS_1D_, and CS_7D_ all have negative Kurtosis values ranging between −0.35 and −1.54, with FLOW having the only positive value of 0.67.

**Figure 2:**
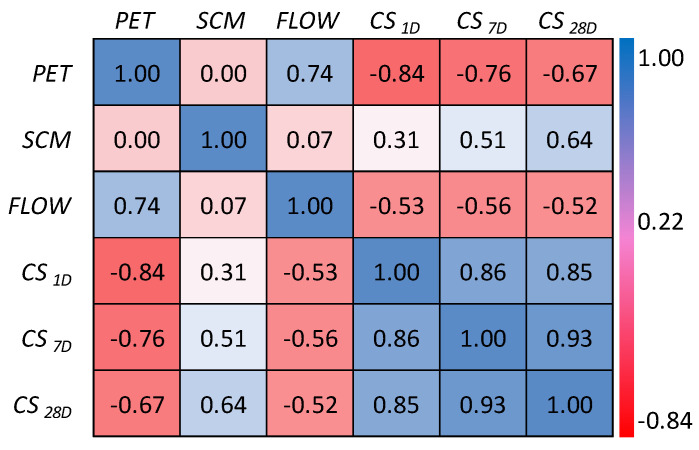
Correlation matrix.

**Figure 3:**
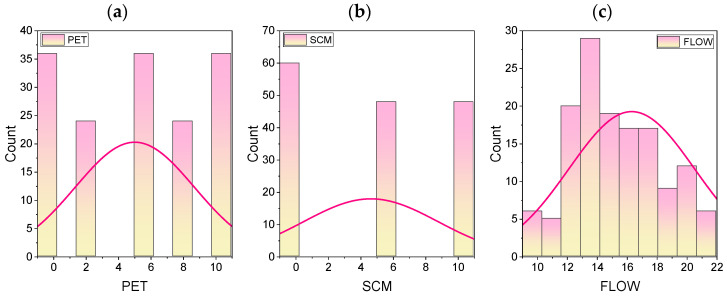

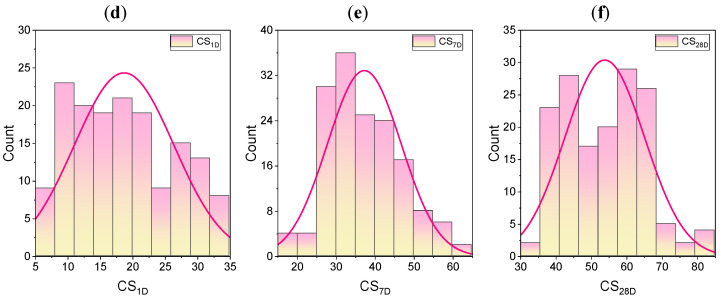
(**a**–**f**) Frequency histogram for the input and output parameters.

To better demonstrate the degree of correlation between the input parameters and CS_28D_, Pearson correlation matrix is presented in Figure 2. From Figure 2, it can be seen that the correlation between CS_1D_ and CS_28D_, and CS_7D_ and CS_28D_ are 0.85 and 0.93, respectively. The correlation between CS_28D_ and other three influencing parameters, i.e., PET, SCM, FLOW, is in the range of −0.67 to 0.64. These data demonstrate that two parameters, i.e., CS_1D_ and CS_7D_, have a significant impact on CS_28D_ of waste PET/ SCM blended cementitious grout. In addition to the correlation matrix, frequency histogram with sample distribution of all the input and output parameters is presented in Figure 3a–f. As can be seen, the parameters SCM, CS_1D_, CS_7D_, and CS_28D_ have a reduced degree of skewness, whereas the parameter FLOW has a very high degree of skewness. However, PET has zero skewness.

### 3.2. Computational Modelling

**Figure 4:**
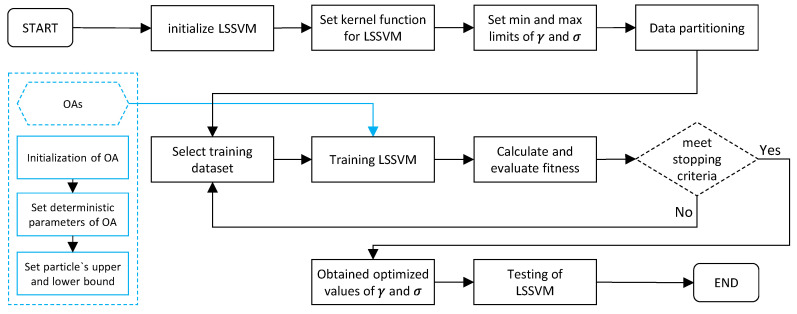
Construction procedure of hybrid LSSVM models.

In this study, the LSSVM hyper-parameter was determined using five MH algorithms. As previously stated, LSSVM has two hyper-parameters, namely γ and σ. It is worth noting that proper setup of these two parameters is necessary for constructing an efficient LSSVM model, due to the fact these two parameters have a significant impact on the performance. Therefore, five MH algorithms, namely PSO, GWO, SMA, and SOS were used to optimize γ and σ, and five hybrid models, i.e., LSSVM-PSO, LSSVM-GWO, LSSVM-SSA, LSSVM-HHO, and LSSVM-SOS, were constructed. For the construction of these models, the following steps were followed: (a) initialize LSSVM and set kernel function; (b) set upper and lower bounds (ub and lb) for γ and σ; (c) set termination criteria and cost function; (d) data partition and selection of training subset; (e) initialize MH algorithms; (f) set different deterministic parameters of MH algorithms such as such as swarm size (N_S_), number of iterations (t_max_), ub, lb, and other parameters; (g) training of LSSVM; (g) calculate fitness in each iteration; (h) check terminating criteria; (i) check fitness and obtained optimum values of γ and σ; (j) generation of hybrid model; and (k) prediction of new dataset using the values of γ and σ. Figure 4 illustrates the hybrid LSSVM development process. Notably, the deterministic parameters of MH algorithms also play a considerable role in hybrid modelling; consequently, they must be calibrated properly during the course of optimization process.

**Figure 5:**
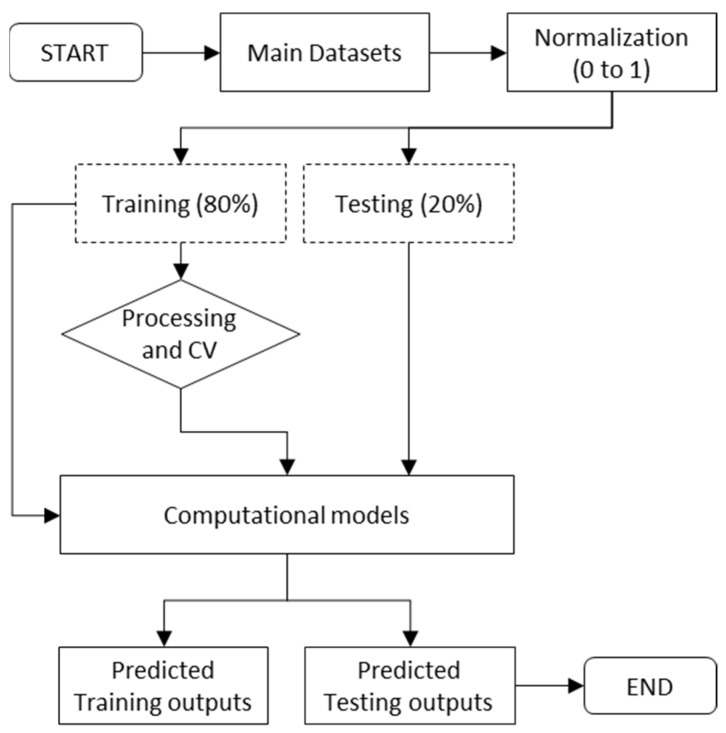
Steps for model construction and validation.

It is pertinent to mention that the main dataset was separated into training and testing subsets for the construction and verification of the hybrid LSSVM models. In this study, 80% of the overall dataset was allocated to the training subset, while the remaining 10% was allocated to the testing subset. Note thatwhen it comes to deciding how many samples to utilize for training a data-driven model, there is no pre-defined criterion available. However, the researchers’ selection will be driven mostly by the nature of the problem at hand. It is a common practice to consider a model that was constructed using a large dataset to be superior to one that was constructed using a relatively modest number of observed data points. With this information in mind, it was decided that the testing dataset would consist of 20% of the main dataset. Figure 5 shows how computational modelling was used to estimate the CS of waste PET/SCM blended cementitious grout. It may be noted that, five-fold cross-validation approach was used to develop the best prediction model.

## 4. Results and Discussion

This section describes the results of the hybrid LSSVM models used to estimate the CS of waste PET/SCM blended cementitious grout. As indicated previously, prior to the creation of the models, the primary dataset was divided into training (125 samples) and testing (31 samples) subsets. All models were constructed and validated using identical training and testing subsets. The outcomes of the constructed LSSVMs were then examined using a number of metrics. In contrast, in addition to γ and σ, the N_S_, t_max_, ub, lb, and other deterministic parameters play an important part in hybrid modelling; thus, they were calibrated appropriately during optimization. This is mainly due to the selection of optimum values of hyper-parameters. The following sub-section describes the configuration of different deterministic and hyper-parameters of hybrid LSSVM models in estimating the CS of waste PET/SCM blended cementitious grout.

### 4.1. Parametric Details

As was already mentioned, choosing the optimum LSSVM hyper-parameters and deterministic parameters of MH are important for constructing the best model. Because of this, the values of γ and σ were set within a range with ub and lb. In this study, the ub and lb of γ and σ were set to (100 and 0.10) and (50 and 0.10), respectively. Based on γ and σ =LB+rand×(UB−LB), the values of these two parameters were chosen at random and minimize the cost function iteratively. Here, rand represents a uniformly distributed random between [0–1].

**Figure 6:**
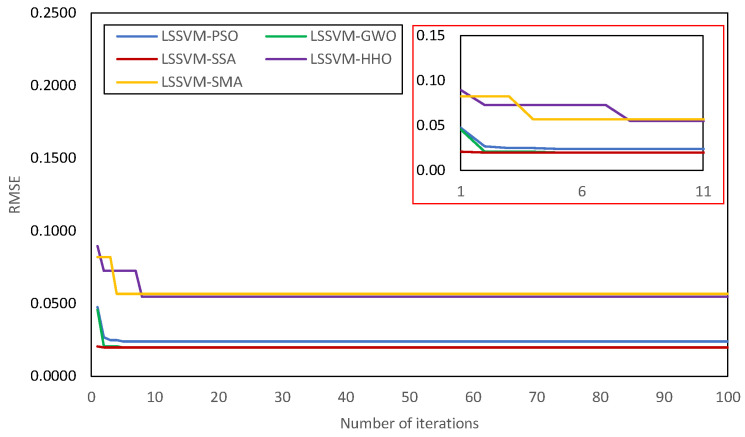
Convergence curve of the developed hybrid LSSVMs.

In each case, the values of N_S_ and t_max_ were set to 30 and 100, respectively, The PSO parameters c_1_ (cognitive coefficient) and c_2_ (social coefficient) were set to 1 and 2, respectively. The value of SMA parameter z, was set to 0.2. Note that the values of the exploration and exploitation constants were kept at their original values for other MH. Table 2 shows the hyper-parameters of the constructed LSSVMs and the deterministic parameters of MH algorithms. In addition, the convergence behavior of the developed hybrid LSSVM models is presented in Figure 6. In the following sub-section, the performance of the LSSVM-PSO, LSSVM-GWO, LSSVM-SSA, LSSVM-HHO, and LSSVM-SMA models is presented and discussed. As indicated previously, the entire dataset was divided into training and testing datasets in the proportions of 80% and 20%, respectively. In order to perform a five-fold cross-validation (CV) technique, the dataset was specifically partitioned 80:20. The best performing model was chosen and examined based on performance gained (in terms of RMSE criterion) throughout the testing phase. Note that, in Figure 6, the convergence behavior of the best performing model has been presented. In Table 3, the performance of five-fold CV is presented. The CV-1 dataset with lowest RMSE value was selected and analysed in the following sub-section.

### 4.2. Model Performance

It is important to note that, right after model development, various performance metrics including Adj.R^2^, NS, PI, R^2^, RMSE, RSR, VAF, and WI, were used to evaluate hybrid LSSVMs. Note that these indices are frequently used [94,95,96,97,98,99,100,101,102,103,104,105,106,107,108,109,110,111,112,113,114,115,116] to evaluate the generalization capabilities of any prediction model from a variety of perspectives, including correlation accuracy, related error, variance, and so on. The expressions of these indices can be given as follows:

(5) $$Adj.R^{2}=1-\frac{\left(n-1\right)}{\left(n-p-1\right)}\left(1-R^{2}\right)$$

(6) $$NS=1-\frac{\sum_{i=1}^{n}{\left(y_{i}-{\hat{y}}_{i}\right)}^{2}}{\sum_{i=1}^{n}{\left(y_{i}-y_{mean}\right)}^{2}}$$

(7) $$PI=adj.R^{2}+0.01VAF-RMSE$$

(8) $$R^{2}=\frac{\sum_{i=1}^{n}{\left(y_{i}-y_{mean}\right)}^{2}-\sum_{i=1}^{n}{\left(y_{i}-{\hat{y}}_{i}\right)}^{2}}{\sum_{i=1}^{n}{\left(y_{i}-y_{mean}\right)}^{2}}$$

(9) $$\mathrm{RMSE}=\sqrt{\frac{1}{n}\sum_{i=1}^{n}{\left(y_{i}-{\hat{y}}_{i}\right)}^{2}}$$

(10) $$RSR=\frac{RMSE}{\sqrt{\frac{1}{n}\sum_{i=1}^{n}{\left(y_{i}-y_{mean}\right)}^{2}}}$$

(11) $$VAF\;\left(\%\right)=\left(1-\frac{var(y_{i}-{\hat{y}}_{i})}{var(y_{i})}\right)\times 100$$

(12) $$WI=1-\left[\frac{\sum_{i=1}^{n}{\left(y_{i}-{\hat{y}}_{i}\right)}^{2}}{\sum_{i=1}^{n}{\left\{\left|{\hat{y}}_{i}-y_{mean}\right|+\left|y_{i}-y_{mean}\right|\;\right\}}^{2}}\right]$$

where *p* and n represent the total number of input parameters and observations, respectively; $y_{i}$ and ${\hat{y}}_{i}$ are the actual and predicted *i*th values, respectively; and $y_{mean}$ is the average of actual value. It is important to note that the value of these indices must match their ideal value for an ideal model, which is provided in Table 4. It should also be noted that multiple indices were used to assess the combined accuracy of the developed hybrid LSSVM models from different aspects such as degree of correlation between the actual and predicted values, associated error, variance, and relative error to the actual/experimental values.

Based on the performance of five-fold CV, the best performing model was selected for prediction. From the information presented in Table 3, it can be shown that the LSSVM-PSO model has a smaller standard deviation for all of the RMSE values, demonstrating its superiority in the testing phase. Because CV-1 dataset has a lower level of error, it was chosen to be the basis for hybrid LSSVM modelling. The performance of the developed hybrid LSSVMs is presented in Table 5, Table 6 and Table 7, respectively, for the training, testing, total datasets. The performance of the model when used to forecast the training and testing datasets is presented in Table 5 and Table 6, respectively. It should be mentioned that the model’s performance using the training dataset was used to express the goodness of fit, while the testing dataset was utilized to validate the predictive capability of the hybrid LSSVM models. Based on R^2^ and RMSE values, the constructed LSSVM-GWO attained the precise prediction (R^2^ = 0.9924 and RMSE = 0.0199) in the training phase. The other models exhibit good agreement with the experimental dataset, with R^2^ values ranging from 0.9397 to 0.9924. Both LSSVM-GWO and LSSVM-SSA achieved the same level of accuracy, as seen by their same NS, PI, R^2^, RMSE, RSR, and WI values.

However, in the validation phase, the LSSVM-PSO was found to be the best performing model with Adj. R^2^ = 0.9649, R^2^ = 0.9708, RMSE = 0.0424, and VAF = 96.9520. From the results presented in Table 7, it can also be observed that that the proposed LSSVM-PSO model achieves the best results in all matrices for the total dataset, with Adj. R^2^ = 0.9842, R^2^ = 0.9847, RMSE = 0.0285, RSR = 0.1243, and VAF = 98.4635. Based on testing results, it was determined that the LSSVM-GWO was the second-best model (Adj. R^2^ = 0.9463, R^2^ = 0.9553, RMSE = 0.0551, RSR = 0.2337, and VAF = 94.7355), followed by LSSVM-SSA model (Adj. R^2^ = 0.9463, R^2^ = 0.9553, RMSE = 0.0551, RSR = 0.2337, and VAF = 94.7350), LSSVM-HHO (Adj. R^2^ = 0.9281, R^2^ = 0.9401, RMSE = 0.0578, RSR = 0.2448, and VAF = 94.0068), and LSSVM-SMA (Adj. R^2^ = 0.9244, R^2^ = 0.9370, RMSE = 0.0602, RSR = 0.2553, and VAF = 93.5441). The results of score analysis also indicate that the proposed LSSVM-PSO attained the most accurate prediction with a total score of 40, followed by LSSVM-GWO (total score = 32), LSSVM-SSA (total score = 24), LSSVM-HHO (total score = 16), and LSSVM-SMA (total score = 8). Overall, the proposed LSSVM-PSO is significantly better than other hybrid LSSVM models and it can be concluded that the PSO is indeed helpful in constructing the hybrid LSSVM model used for prediction of CS_28D_ of waste PET and SCM blended cementitious grout.

### 4.3. Discussion of Results

**Figure 7:**
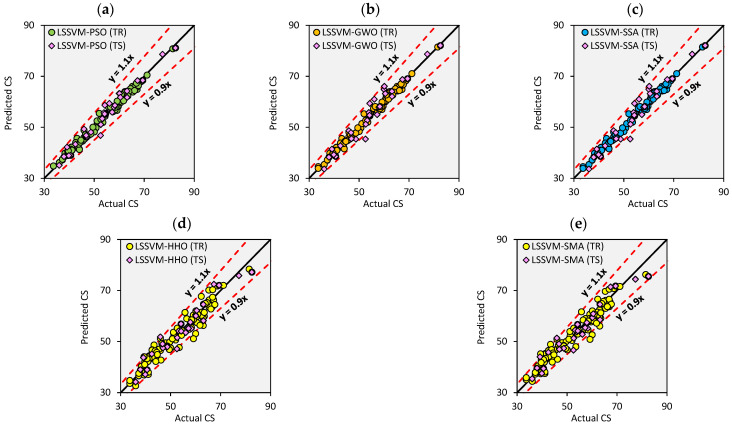
(**a**–**e**) Scatter plots between actual and predicted CS values for the training and testing datasets.

In order to complete a data-driven study, the results and statistical ramifications must be visualized. The use of visualizations aids in the detection of trends, patterns, noise, and outliers in datasets that are more readily understood by the human brain. When wading through a large and unorganized dataset, it is time-consuming to read the actual dataset. Thus, the results of the constructed hybrid LSSVM are shown graphically in this sub-section. Illustration of scatterplots between the actual and predicted CS of waste PET and SCM blended cementitious grout is presented in Figure 7. Herein, combined scatterplots for the training and testing datasets are presented. As noted, the dispersion of all created models falls inside the ± 10% deviation line, confirming their preciseness during both the training and testing phases.

**Figure 8:**
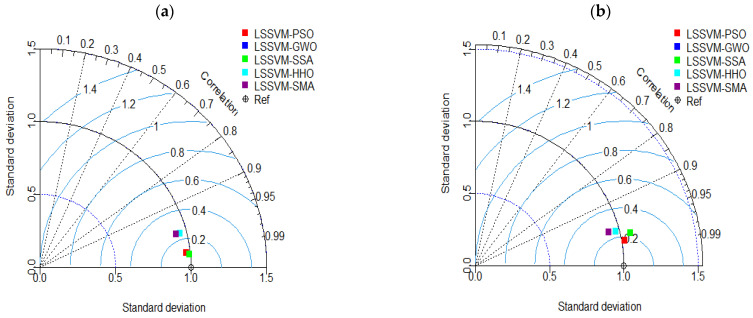
Taylor diagrams for the training (**a**) and testing (**b**) datasets.

Furthermore, a Taylor diagram [117] and error plots are presented to visualize the overall accuracy comprehensively. Note that Taylor diagrams are useful for quickly evaluating the precision of a model because of the brief information they provide in a 2D mathematical diagram. Correlation coefficient, RMSE, and ratio of standard deviations are all measures used to describe the preciseness between the experimental and estimated observations. In a Taylor diagram, a model is represented by a “point.” It is important to highlight that in a perfect model, the location of the “point” would be identical to the location of the “reference point” (as shown in black color “Ref” point). Taylor diagrams for the training and testing outcomes are shown in Figure 8, providing a clear illustration of the accuracy of the created LSSVM-PSO in the testing phase.

**Figure 9:**
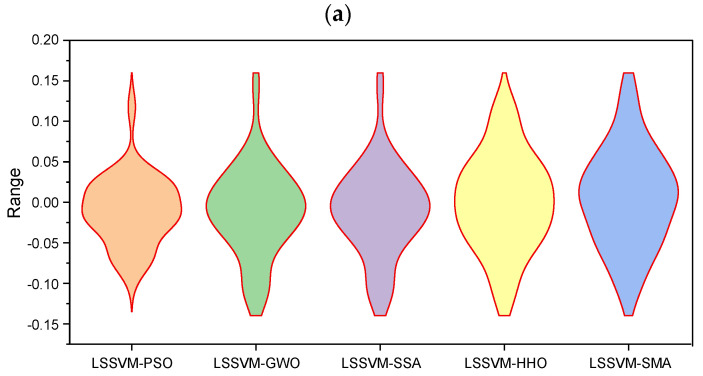

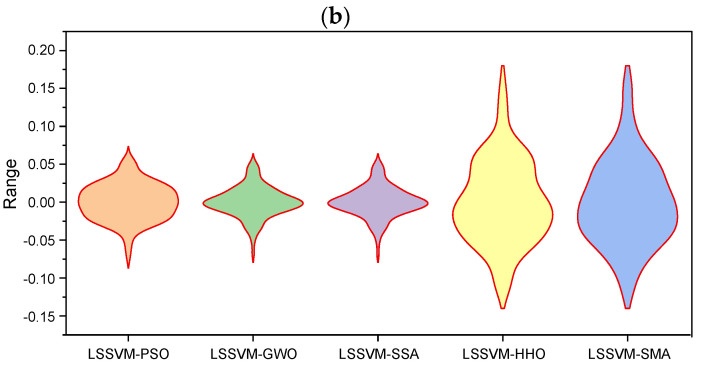
Error plot for the training (**a**) and testing (**b**) datasets.

Alternatively, the box plot of error between the experimental and estimated CS is presented in Figure 9 for both training and testing results. Using this error graphic, one can immediately examine the amount of association of the developed models. As stated previously, multiple performance indices must be established to examine the preciseness of a model from various perspectives; however, interpreting findings by studying the values of each parameter is not only time-consuming, but also requires extensive observations. Thus, illustration of results in the form of scatterplot, Taylor diagram and error plot, is highly beneficial for rapid evaluation of a data-driven model.

From the information presented in Table 5, Table 6 and Table 7 and Figure 7, Figure 8 and Figure 9, it can be deduced that the developed LSSVM-PSO attained the most precise prediction in the testing phase, indicating its superiority in predicting the CS_28D_ of waste PET and SCM blended cementitious grout. However, to better to demonstrate the generalization capability of the proposed LSSVM-PSO, sensitivity analysis (SA) was conducted to determine the effect of various input parameters on the output. Additionally, OBJ creation was used to evaluate the overall preciseness of the proposed LSSVM-PSO model.

In this study, a commonly used cosine amplitude approach [118] was employed to quantify the relative impact of PET, SCM, FLOW, CS_1D_, and CS_7D_ on the output, i.e., CS_28D_. The outcomes of SA are shown in Table 8. The effect of PET, SCM, FLOW, CS_1D_, and CS_7D_ on the output is provided exclusively for LSSVM-PSO, LSSVM-GWO, LSSVM-SSA, LSSVM-HHO, and LSSVM-SMA models. Note that the value close to unity signifies that the parameter has the higher influence on the output. SA also displays the accuracy with which a data-driven model can predict the desired outcome based on the influence of inputs on output as determined by the original experimental results. On the other hand, OBJ creation is used to establish how well a data-driven model works as a whole by combining the R^2^ and MAE values obtained in both training and testing phases. The following expression is used to determine OBJ creation value [119].

(13) $$OBJ=\left(\frac{N_{TR}-N_{TS}}{N_{TL}}\right)\times \left(\frac{MAE_{TR}}{{R^{2}}_{TR}}\right)+\left(\frac{2\times N_{TR}}{N_{TL}}\right)\times \left(\frac{MAE_{TS}}{{R^{2}}_{TS}}\right)$$

where $N_{TR}$, ${R^{2}}_{TR}$, and $MAE_{TR}$ are the number of samples, determination coefficient, and MAE values, respectively, for the training dataset, and $N_{TS}$, ${R^{2}}_{TS}$ and $MAE_{TS}$ are also indicate the same parameters, bur for the testing dataset.

The values OBJ creation for all the developed hybrid models are presented in Table 9. Furthermore, all the models were ranked based on OBJ value. It can be seen that the developed LSSVM-PSO secured first rank in estimating the CS_28D_ of waste PET and SCM blended cementitious grout. The developed LSSVM-GWO was determined the second-best model followed by LSSVM-SSA, LSSVM-HHO, and LSSVM-SMA.

In comparison, Khan et al. [56] conducted a detailed study on changing genetic parameters of GEP model. The GEP model that was ultimately chosen had the best statistical indices for the training and validation datasets, respectively (R = 0.977 and 0.975, RMSE = 2.423 and 2.531, MAE = 1.918 and 2.055). It was established that for achieving sustainable and green concrete, the function of PET/SCM has no detrimental effects on the CS_28D_ of cementitious grouts, making PET a viable option. The straightforward mathematical formulation of GEP was found to be effective, which results in time savings and lower personnel costs for testing in civil engineering projects. The performance of the currently developed models is comparable to the previous model, thus are reliable and can be used for accurate prediction of compressive strength of PET incorporated cementitious composites.

## 5. Summary and Conclusions

This study presents a hybrid ML paradigm of LSSVM for estimating the CS_28D_ of waste PET and SCM blended cementitious grout. Two SCMs, namely FA and SF, were mixed with waste PET for this purpose, and cementitious grout was made and tested in the laboratory. Specifically, CS of 156 samples of 50 mm × 50 mm × 50 mm cubes were tested. Subsequently, three influencing parameters, namely PET, SCM, and FLOW, along with CS_1D_ and CS_7D_ were used to estimate the CS_28D_ of cementitious grout using five hybrid LSSVM models. Based on the experimental results with R^2^ and RMSE criteria, the following conclusions can be drawn.

(a) The constructed LSSVM-PSO model attained the most accurate prediction (R^2^ = 0.9708 and RMSE = 0.0424) during the testing phase. Furthermore, the SA and OBJ creation results show that the suggested LSSVM-PSO has achieved the highest level of performance, indicating its robustness at all levels.
(b) The sensitivity analysis revealed that CS_7D_ is most significant parameter which impacts the long term CS_28D_ of cementitious grouts mixes followed by CS_1D_, proportion of the SCM, flow, and the content of PET.
(c) The developed LSSVM-PSO secured first rank in predicting the CS_28D_ of cementitious grout. Additionally, the suggested model has superiority as evidenced by its quicker convergence (within six iterations).
(d) The primary advantage of the constructed LSSVM-PSO model is that the optimized hyper-parameters are transferred to the co-ordination of each particle of the swarm, and each particle’s position in the swarm is a solution for the said model. Since swarm sizes of 30 and 100 iterations were used, only 3000 solutions were analysed in order to acquire the appropriate LSSVM hyper-parameter values.
(e) Furthermore, convergence behaviour reveals that the created LSSVM-PSO model converge within 10 iteration count, showing involvement of very minimal computing effort to reach the specified accuracy level. This is another major advantage of the LSSVM-PSO model.
(f) However, one of the limitations of the proposed model is the limitation of particle position by the search space determined by the PSO parameters. Because there is no rule of thumb, a trial-and-error strategy must be conducted to determine the optimal searching space, which is a time-consuming task. Furthermore, while this study was based on a real-life experimental dataset, the variance in the influencing parameters may not be multi-dimensional. Therefore, more large-scale research should be conducted to expand the use of LSSVM-PSO model in estimating the intended output.
(g) Based on these facts, the proposed LSSVM-PSO model can be utilized as a novel alternative for estimating the CS of cementitious grouts. Despite these limitations, the suggested LSSVM-PSO model offers a new alternative tool for estimating CS prediction of cementitious grouts in many construction projects.

Nonetheless, the future directions of the current study could involve by applying the hybrid framework of PSO and other ML models, such as ANN, ELM, and ANFIS, to the solution of other engineering problems, such as the prediction of the CS of various concrete materials. As per the authors’ knowledge, this is the first research to estimate the CS of PET and SCM blended cementitious grout using hybrid LSSVM models.

## Figures and Tables

**Table 1:** Description details of the main dataset.

Index	PET	SCM	FLOW	CS_1D_	CS_7D_	CS_28D_
Count	156	156	156	156	156	156
Minimum	0.00	0.00	9.10	5.64	19.19	33.64
Mean	5.00	4.62	16.30	18.66	37.20	53.74
Median	5.00	5.00	15.40	17.91	36.54	54.18
Mode	10.00	0.00	14.00	28.22	37.54	57.91
Range	10.00	10.00	19.50	27.67	42.62	48.90
Maximum	10.00	10.00	28.60	33.32	61.81	82.54
Standard error	0.29	0.33	0.34	0.61	0.76	0.90
Standard deviation	3.68	4.16	4.20	7.67	9.47	11.26
Sample variance	13.55	17.27	17.62	58.81	89.62	126.89
Kurtosis	−1.35	−1.54	0.67	−1.07	−0.35	−0.58
Skewness	0.00	0.15	0.95	0.23	0.46	0.30

**Table 2:** Details of different parametric for hybrid LSSVMs.

Parameters	LSSVM-PSO	LSSVM-GWO	LSSVM-SSA	LSSVM-HHO	LSSVM-SMA
N_S_	30	30	30	30	30
t_max_	100	100	100	100	100
$c_{1},c_{2}$	1,2	-	-	-	-
z (Parameter of SMA)	-	-	-	-	0.20
ub and lb for γ	100 and 0.10	100 and 0.10	0 and 0.10	100 and 0.10	100 and 0.10
ub and lb for σ	50 and 0.10	50 and 0.10	and 0.10	50 and 0.10	50 and 0.10
ub and lb for OAs	+1 and −1	+1 and −1	+1 and −1	+1 and −1	+1 and −1

**Table 3:** Performance of CV based on RMSE criterion for the testing dataset.

CV Level	LSSVM-PSO	LSSVM-GWO	LSSVM-SSA	LSSVM-HHO	LSSVM-SMA
CV-1	0.0424	0.0551	0.0551	0.0578	0.0602
CV-2	0.0430	0.0446	0.0446	0.0612	0.0513
CV-3	0.0437	0.0575	0.0575	0.0652	0.0679
CV-4	0.0453	0.0460	0.0460	0.0662	0.0653
CV-5	0.0430	0.0460	0.0460	0.0670	0.0710
Standard deviation	0.0010	0.0053	0.0053	0.0035	0.0069

**Table 4:** Ideal values of different indices.

Name of Different Indices	Abbreviation	Ideal Value
Adjusted coefficient of determination	Adj.R^2^	1
Nash–Sutcliffe efficiency	NS	1
Performance index	PI	2
Coefficient of determination	R^2^	1
Root mean square error	RMSE	0
RMSE to observation’s standard deviation ratio	R	0
Variance account factor	VAF	100
Willmott’s index of agreement	WI	1

**Table 5:** Performance parameters for the training dataset.

Models/Particulars		Adj.R^2^	NS	PI	R^2^	RMSE	RSR	VAF	WI	Total Score
LSSVM-PSO	Value	0.9889	0.9890	1.9541	0.9894	0.0239	0.1050	98.8981	0.9972	24
	Score	3	3	3	3	3	3	3	3	
LSSVM-GWO	**Value**	**0.9921**	**0.9923**	**1.9645**	**0.9924**	**0.0199**	**0.0875**	**99.2349**	**0.9981**	**40**
	**Score**	**5**	**5**	**5**	**5**	**5**	**5**	**5**	**5**	
LSSVM-SSA	Value	0.9920	0.9923	1.9645	0.9924	0.0199	0.0875	99.2347	0.9981	32
	Score	4	4	4	4	4	4	4	4	
LSSVM-HHO	Value	0.9397	0.9419	1.8268	0.9422	0.0548	0.2410	94.1911	0.9846	16
	Score	2	2	2	2	2	2	2	2	
LSSVM-SMA	Value	0.9372	0.9379	1.8184	0.9397	0.0567	0.2492	93.7924	0.9831	8
	Score	1	1	1	1	1	1	1	1	

**Table 6:** Performance parameters for the testing dataset.

Models/Particulars		Adj.R^2^	NS	PI	R^2^	RMSE	RSR	VAF	WI	Total Score
LSSVM-PSO	**Value**	**0.9649**	**0.9677**	**1.8921**	**0.9708**	**0.0424**	**0.1797**	**96.9520**	**0.9920**	**40**
	**Score**	**5**	**5**	**5**	**5**	**5**	**5**	**5**	**5**	
LSSVM-GWO	Value	0.9463	0.9454	1.8386	0.9553	0.0551	0.2337	94.7355	0.9871	32
	Score	4	4	4	4	4	4	4	4	
LSSVM-SSA	Value	0.9463	0.9454	1.8386	0.9553	0.0551	0.2337	94.7350	0.9871	24
	Score	3	3	3	3	3	3	3	3	
LSSVM-HHO	Value	0.9281	0.9401	1.8104	0.9401	0.0578	0.2448	94.0068	0.9844	16
	Score	2	2	2	2	2	2	2	2	
LSSVM-SMA	Value	0.9244	0.9348	1.7996	0.9370	0.0602	0.2553	93.5441	0.9822	8
	Score	1	1	1	1	1	1	1	1	

**Table 7:** Performance parameters for the total dataset.

Models/Particulars		Adj.R^2^	NS	PI	R^2^	RMSE	RSR	VAF	WI	Total Score
LSSVM-PSO	**Value**	**0.9842**	**0.9846**	**1.9403**	**0.9847**	**0.0285**	**0.1243**	**98.4635**	**0.9961**	**40**
	**Score**	**5**	**5**	**5**	**5**	**5**	**5**	**5**	**5**	
LSSVM-GWO	Value	0.9824	0.9825	1.9346	0.9829	0.0303	0.1322	98.2613	0.9957	32
	Score	4	4	4	4	4	4	4	4	
LSSVM-SSA	Value	0.9824	0.9825	1.9346	0.9829	0.0303	0.1322	98.2610	0.9957	24
	Score	3	3	3	3	3	3	3	3	
LSSVM-HHO	Value	0.9400	0.9418	1.8263	0.9419	0.0554	0.2413	94.1751	0.9846	16
	Score	2	2	2	2	2	2	2	2	
LSSVM-SMA	Value	0.9373	0.9375	1.8174	0.9393	0.0574	0.2500	93.7544	0.9829	8
	Score	1	1	1	1	1	1	1	1	

**Table 8:** Results of SA for the total dataset.

Parameters	Actual	LSSVM-PSO	LSSVM-GWO	LSSVM-SSA	LSSVM-HHO	LSSVM-SMA
PET	0.5111	0.5146	0.5084	0.5084	0.5134	0.5200
SCM	0.8570	0.8575	0.8561	0.8561	0.8620	0.8625
FLOW	0.6272	0.6313	0.6261	0.6261	0.6353	0.6443
CS_1D_	0.9620	0.9647	0.9639	0.9639	0.9709	0.9709
CS_7D_	0.9844	0.9871	0.9865	0.9865	0.9914	0.9904

**Table 9:** Details of OBJ creation estimation.

Models	MAE TR	MAE TS	R^2^ TR	R^2^ TS	OBJ_1	OBJ_2	OBJ	Rank
LSSVM-PSO	0.0193	0.0328	0.9894	0.9708	0.0117	0.0134	0.0252	1
LSSVM-GWO	0.0144	0.0406	0.9924	0.9553	0.0088	0.0169	0.0257	2
LSSVM-SSA	0.0144	0.0406	0.9924	0.9553	0.0088	0.0169	0.0257	3
LSSVM-HHO	0.0437	0.0466	0.9422	0.9401	0.0279	0.0197	0.0476	4
LSSVM-SMA	0.0448	0.0479	0.9397	0.9370	0.0287	0.0203	0.0490	5

## Data Availability

The data used in this research has been properly cited and reported in the main text.
